# Effects of different intracranial volume correction methods on univariate sex differences in grey matter volume and multivariate sex prediction

**DOI:** 10.1038/s41598-020-69361-9

**Published:** 2020-07-31

**Authors:** Carla Sanchis-Segura, Maria Victoria Ibañez-Gual, Naiara Aguirre, Álvaro Javier Cruz-Gómez, Cristina Forn

**Affiliations:** 1 Departament de Psicologia Bàsica, Clínica i Psicobiologia, Universitat Jaume I, Avda. Sos Baynat, SN, 12071 Castelló, Spain; 2 Department of Mathematics, IMAC, Universitat Jaume I, Castelló, Spain

**Keywords:** Sexual dimorphism, Brain

## Abstract

Sex differences in 116 local gray matter volumes (GM_VOL_) were assessed in 444 males and 444 females without correcting for total intracranial volume (TIV) or after adjusting the data with the scaling, proportions, power-corrected proportions (PCP), and residuals methods. The results confirmed that only the residuals and PCP methods completely eliminate TIV-variation and result in sex-differences that are “small” (∣*d*∣ < 0.3). Moreover, as assessed using a totally independent sample, sex differences in PCP and residuals adjusted-data showed higher replicability ($\approx$ 93%) than scaling and proportions adjusted-data $( \approx$ 68%) or raw data ($\approx$ 45%). The replicated effects were meta-analyzed together and confirmed that, when TIV-variation is adequately controlled, volumetric sex differences become “small” (∣*d*∣ < 0.3 in all cases). Finally, we assessed the utility of TIV-corrected/ TIV-uncorrected GM_VOL_ features in predicting individuals’ sex with 12 different machine learning classifiers. Sex could be reliably predicted (> 80%) when using raw local GM_VOL_, but also when using scaling or proportions adjusted-data or TIV as a single predictor. Conversely, after properly controlling TIV variation with the PCP and residuals’ methods, prediction accuracy dropped to $\approx$ 60%. It is concluded that gross morphological differences account for most of the univariate and multivariate sex differences in GM_VOL_

## Introduction

The study of neuroanatomical sex differences in the brain is a subject of considerable scientific importance^1–3^ that also arouses great interest in the popular press and lay audiences^4,5^. However, precisely quantifying sex differences in the volumes of specific brain regions is a challenging task that is severely complicated by the existing sex differences in the overall body and head size^6–8^. Thus, although there is an increasing consensus about the need to parse out the quantitative contribution of “direct” or “specific” effects of sex on regional brain volumes from those derived from gross morphology differences between females and males^9–18^, there is far less agreement about how to do this. Thus, several adjusting variables (for a review, see O’Brien, 2006) and statistical methods are currently used to adjust gross morphology variation^7,8,15,16^.

Within this context, we recently conducted a broad systematic study to compare how five different TIV-adjustment methods (scaling as implemented by the non-linear modulation option of the VBM8 toolbox, proportions, power-corrected-proportions, covariate regression, or the residuals methods) affected the number, size, and direction of sex differences in 116 local gray matter volumes (GM_VOL_) in the so-called “UJI-sample”^19^. Our results confirmed and extended those of other previous studies by showing that: (1) males have larger TIV-uncorrected (raw) GM_VOL_ in all brain areas, but these differences are largely due to TIV-variation^9,10,13,14,17^; (2) different TIV-adjustment methods end up producing different patterns of sex differences that are not equally valid^15,16^. Regarding the latter, we observed that the scaling and proportions adjustment methods inverted, but did not eliminate, the preexisting relationships between TIV and local GM_VOL_, thus resulting in larger adjusted volumes in females than in males and promoting sex differences that were very distinct in number, size, and direction from those observed in a subgroup of females and males matched on their TIV^19^. Conversely, data adjusted with the three other methods had no influence of TIV-variation and resulted in fewer, smaller, and bi-directional sex differences that closely resembled those observed in the sample of TIV-matched males and females^19^.

The first aim of the present study was to confirm these results by directly replicating them in a larger sample (hereinafter referred as the “HCP sample”) composed of 444 females and 444 males. Replication of findings is a cornerstone of scientific progress because it makes it possible to increase the precision of effect size estimates and provide information about whether an earlier published effect should be considered a true effect, a false positive, or the result of an interaction with a contextual moderator^20,21^. Replication should not be assessed based on coincidence analyses of “significant/non-significant effects”^22–24^, but on other metrics specifically developed to compare the effects found in different samples from a single population (i.e. prediction intervals^23,25^). Therefore, in the present study, prediction intervals were calculated to assess the extent to which the direction and size (Cohen’s *d* values) of the sex differences in GM_VOL_ obtained in the present study replicated the ones we previously observed in the “UJI-sample”^19^.

This replication assessment also allowed us to address a largely unexplored question, namely, the extent to which the replicability of sex differences in GM_VOL_ is affected by the method employed to adjust TIV-variation. In this regard, although TIV-adjustment is known to increase measurement error and reduce the reliability of local GM_VOL_ measurements^26,27^, it actually improves the detection of between-group differences in GM_VOL_^27^. Moreover, random measurement error in TIV values has been found to increase variability and reduce between-groups mean differences in proportions-adjusted data, but not in residuals-adjusted data^28^. Therefore, it can be tentatively hypothesized that at least some TIV-adjustment methods could increase the replicability of sex differences in GM_VOL_, especially when considering the replicability of effect sizes based on means and standard deviations, such as Cohen’s *d*. However, to our knowledge, this proposal has not been empirically tested.

As a third and final objective, in the present study we also explored the effect of different TIV-adjustment methods when assessing multivariate sex differences. Assessing multivariate sex differences is important because a series of small univariate differences might (or might not) aggregate into a larger overall difference, and because multivariate statistics provide non-redundant, distinct information from what univariate measures convey^29–31^. Multivariate sex differences can be assessed through effect size indexes such as Mahalanobis’ *D* (the multivariate equivalent of Cohen’s *d;* see^29,30^). However, *D* and other related effect sizes are more meaningful when summarizing a “*coherent, theoretically justified set of variables*”^31^ p. 11) than when comparing whole-brain averages of local effects running in disparate directions (for a detailed discussion, see^31^). Alternatively, multivariate differences can be investigated through classification/prediction statistical techniques collectively referred to as machine learning or statistical learning^32–34^. These techniques are increasingly being used in the study of brain sex differences^31,35–41^ because they make it possible to estimate the degree of statistical distinctiveness or separateness of the brains of females and males at the multivariate level, with the added conceptual appeal of focusing on individual scores instead of on score summaries such as means. However, to our knowledge, no previous study has specifically analyzed to what extent this multivariate distinctiveness is affected by TIV-adjustment.

Therefore, in the present study, we assessed how four currently used TIV-adjustment methods affect the collective utility of 116 local GM_VOL_ when inputed as features of 12 different machine learning algorithms constructed to differentiate the brains of females and males and predict individuals’ sex. Following current recommendations, classification algorithms were fitted, tested, and validated in separate groups of participants^33,34^. More specifically, each algorithm was initially fitted in a randomly selected *training subsample* that comprised 311 females and 311 males (70% of total) from the HCP-sample. The obtained classifiers were internally validated^34^ in the *testing subsample*, which was composed of the hold-out participants from the HCP sample (133 females and 133 males). Finally, the classification algorithms were externally validated^34^ in a completely independent sample (the so-called *external validation subsample*)*,* composed of 171 females and 171 males of the UJ- sample).

## Results and discussion

### Univariate sex differences in the HCP sample

#### Raw data

**Figure 1:**
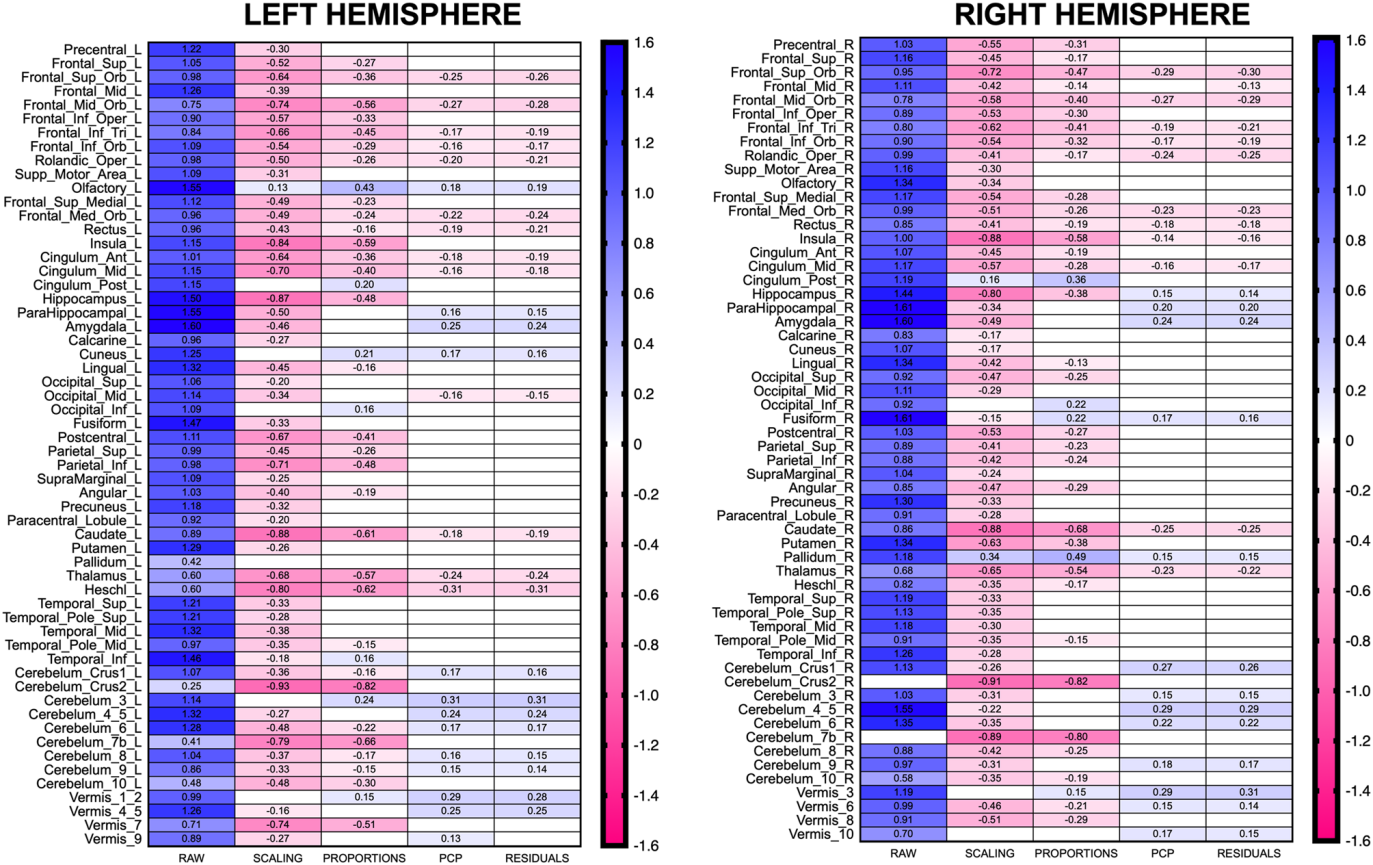
Size and location of sex differences in each dataset of the HCP sample. Panels left and right present odd- and even-numbered brain anatomical regions of the AAL atlas, which (with the exception of the lobules of the cerebellar vermis) are located in the left and right hemisphere, respectively. Heatmaps display the Cohen’s *d* values for statistically significant sex differences (a more detailed description of all effects is provided in Supplementary Tables 1A–5A). Blue colored cells and positive *d* values correspond to M > F effects, whereas red colored cells and negative *d* values correspond to F > M effects.

Males had larger TIVs than females (Difference in means: 212.80 ml, 95% CI [197.73, 227.86]; *d* = 1.86, *p* = 2.65^–122^). Males also exhibited larger raw GM_vol_ than females in the 116 anatomical regions considered in the present study. In 114 cases (98.28% of the total), the confidence intervals for these differences did not include the zero value, making it possible to reject a null hypothesis of no difference between means (uncorrected *p*-values range: 0.0002—2.26^–98^). As Fig. 1 depicts, in these 114 VOIs, Cohen’s *d* values ranged from 0.25 (#93, Cerebellum_Crus2_L; overlap = 90.0%, PS = 57.0%) to 1.61 (#40, Parahippocampal_R; overlap = 42.1%, PS = 87.3%); average *d* = 1.06, 95%CI [0.99, 1.10]. For more detailed information about the sex differences observed in this data set, see Supplementary Table 1A.

**Figure 2:**
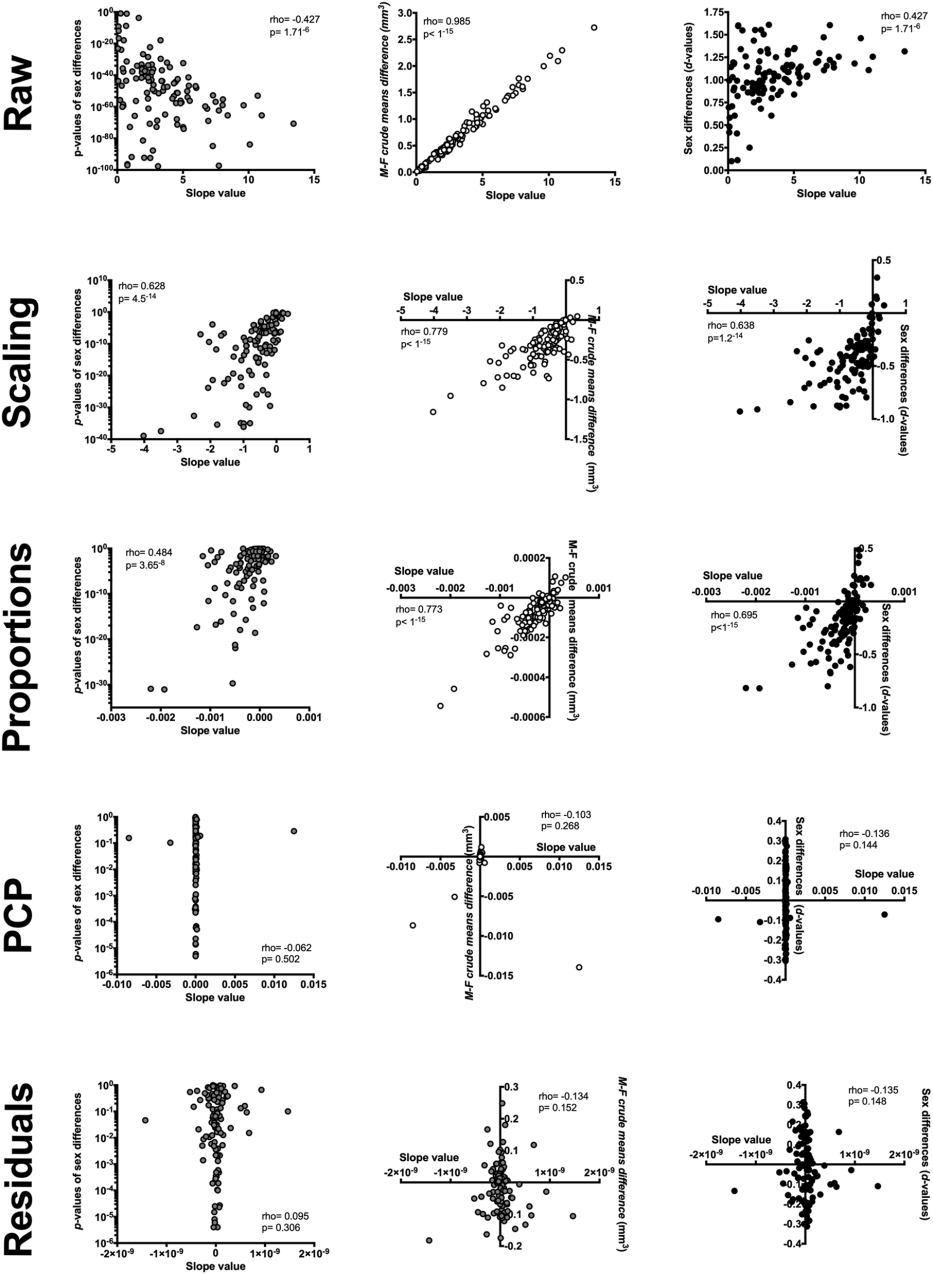
Correlation between TIV-VOI slopes and observed sex differences in each data set. Ordinal correlations (Spearman’s rho) were calculated between the slope values of the TIV-VOI regression lines (provided in Supplementary Tables 1B–5B) and the *p*-values (left column), unstandardized means difference (central column), and Cohen’s *d* values (right column) of the sex differences obtained in the raw, scaling, proportions, PCP, and residuals datasets. Note that the scales and labels’ positioning are customized in each figure to better show the very distinct patterns of correlations observed in each dataset.

TIV variation was directly related to GM_vol_ variation in the 116 VOI_raw_ considered in the present study (*p*-values < 1.81^–5^ in all cases; Supplementary Table 1B). The percent of variance accounted for by TIV differed at each VOI, ranging between 2.05% (#94, Cerebellum_Crus2_R) and 73.59% (#56, Fusiform_gyrus_R). As Fig. 2 shows, the slope values of these TIV-VOI_raw_ linear regressions were correlated with the *p*-values (rho = -0.43, p < 1.7^–6^) and the size (unstandardized mean difference, rho = 0.99, p < 1^–15^; *d*-values, rho = 0.42, p < 1.7^–6^) of the sex differences observed in raw GM_vol_. These results confirm that the significance levels, size, and direction of the sex differences in raw GM_vol_ are largely dependent on TIV variation.

#### Scaled data

On the one hand, females exhibited larger scaled GM_vol_ (GM_scaling_) than males in most of the VOIs. In 107 cases (92.24% of total), the 95% CI of these differences did not include the zero value, allowing to reject the null hypothesis of no differences between means (uncorrected *p*-values range: 0.046 to 1.21^–39^). As Fig. 1 shows, F > M differences were observed in 104 of these 107 VOIs, with *d* values ranging from -0.15 (#56, Fusiform_R; overlap = 93.9%; PS = 54.3%) to -0.93 (#93, Cerebellum_Crus2_L; overlap = 64.2%; PS = 74.5%); average *d* = − 0.47, CI [− 0.43, − 0.50]. On the other hand, males had larger GM_scaling_ in the Olfactory_L (#21; *d* = 0.13, overlap = 94.6%; PS = 53.8%), Cingulum_post_R (#36; *d* = 0.15, overlap = 93.8%; PS = 54.4%), and Pallidum_R (#76; *d* = 0.34, Overlap = 86.7%; PS = 59.4%). For more detailed information about the sex differences observed in this data set, see Supplementary Table 2A.

The scaling method reduced and, in most cases, inverted the direction, but it did not eliminate the effects of TIV on GM_vol_ variation (Supplementary Table 2B). Thus, in 90 cases, TIV-VOI_scaling_ linear regressions had slope values that were statistically different from 0 (*p*-values ranging from 0.047 to < 1^–15^), with TIV explaining percentages of variance ranging between 0.45% (#53, Occipital_inf_L) and 22.44% (#94, Cerebellum_Crus2_R). As Fig. 2 reveals, the slope values of the 116 regression TIV-VOI_scaling_ lines were correlated with the *p*-values (rho = 0.63, p = 4.5^–14^) and the size (unstandardized mean difference, rho = 0.78, p < 1^–15^; *d* values, rho = 0.64, p = 1.2^–14^) of the observed sex differences in GM_scaling._

#### Proportions-adjusted data

As in the case of GM_scaling,_ proportions-adjusted GM_vol_ (GM_prop_) were larger in females than in males. In 76 cases (65.51% of total), the 95%CI of these differences did not include the zero value and allowed to reject the null hypothesis of no difference between means (uncorrected *p*-values range: 0.045—9.6^–32^). In 64 of these 76 cases (84.2%), females exhibited larger GM_prop_ than males, with Cohen’s *d* values ranging from − 0.13 (#48, Lingual_R) to − 0.82 (#94, Cerebellum_Crus2_R); average *d* = − 0.35, CI [− 0.39, − 0.30]. These *d* values (depicted in Fig. 1) translated into degrees of overlap ranging between 68.2% and 94.7%, and PS ranging between 53.8% and 71.9%. In the 12 cases where males had larger GM_prop_ than females, differences ranged from *d* = 0.15 (#109, vermis_1_2; overlap = 94.1%; PS = 54.2%) to *d* = 0.49 (#76, pallidum_R; overlap = 80.8%, PS = 63.5%). For more detailed information about the sex differences observed in this data set, see Supplementary Table 3A.

The proportions method reduced and, in most cases, inverted but did not eliminate the effects of TIV on GM_VOL_ variation (Supplementary Table 3B). In 77 anatomical regions, the slope values for linear TIV-VOI_prop_ were significantly different from zero (*p-*values range: 0.03 to 1^–15^) and, at these VOIs, TIV explained between 0.5% (#85, Temporal_Mid_L) and 24.76% (#94, Cerebellum_Crus2_R) of the observed variance. As Fig. 2 shows, the slope values of the 116 TIV-VOI_prop_ linear relationships were correlated with *p*-values (rho = 0.482, p = 3.65^–8^) and the size (unstandardized mean difference, rho = 0.77, p < 1^–15^; *d* values, rho = 0.65, p < 1^–15^) of the sex differences observed in GM_prop_ (Fig. 2).

#### PCP-adjusted data

Sex differences in PCP-adjusted GM_vol_ (GM_PCP_) showed a clearly bidirectional pattern. In 50 VOIs, the 95% CI of the between-means difference did not include the zero value and allowed to reject the null hypothesis (uncorrected *p*-values range: 0.047 to 5.0^–6^). Within this subset (depicted in Fig. 1), there were 26 M > F differences, with *d* values ranging between 0.11 (#115, Vermis_9; overlap = 94.7%, PS = 53.8%) and 0.31 (#95, Cerebellum_3_L; overlap = 87.8%; PS = 58.6%); average *d* = 0.20, CI [0.18, 0.23]. In addition, F > M differences were observed in 24 brain anatomical regions (Fig. 1; Supplementary Table 4A). In these 24 cases, *d* values ranged between − 0.14 (#30, Insula_R; overlap = 94.3%, PS = 54.0%) and − 0.31 (#79, Heschl_L; overlap = 87.9%; PS: 58.4%); average *d* = − 0.21, CI [− 0.19, − 0.23]. For more detailed information about the sex differences observed in this dataset, see Supplementary Table 4A.

Sex differences in GM_PCP_ were devoid of any influence of TIV. Linear regression analyses indicated that TIV did not account for any of the variance observed in the 116 VOIs in this dataset (r^2^ values ranged between 2.19^–4^ and 5.57^–12^; *p*-values > 0.66 in all cases). All the slope values were also virtually zero (absolute values ranging from 0.01 to 2.79^–9^; Supplementary Table 4B), and, consequently, they were uncorrelated with the *p*-values (rho = − 0.06, p = 0.502) and the size (unstandardized mean difference, rho = − 0.10, p = 0.268; *d* values, rho = − 0.14, p = 0.144) of the sex differences observed in GM_PCP_ (Fig. 2).

#### Residuals-adjusted data

Sex differences in the residuals-adjusted GM_vol_ (GM_res_) were very similar to those observed in the GM_PCP_. In 50 cases, the CIs for these differences did not include the zero value and allowed us to reject the null hypothesis of no differences between means (uncorrected *p*-values ranging from 0.046 to 4^–6^). On the one hand, females had larger local GM_res_ in 25 VOIs_,_ with *d* values ranging from − 0.13 (#8, Frontal_Mid_R: overlap = 94.7%; PS = 53.8%) to -0.32 (#79, Heschl_L; overlap = 87.6% PS = 58.8%); average *d* = − 0.22, CI [− 0.20, − 0.24]. On the other hand, in the 25 anatomical regions in which males had larger GM_res_, *d* values ranged from to 0.14 (#105 Cerebellum_9_L; overlap = 94.5%; PS = 53.9%) to 0.31 (#95, Cerebellum_3_L; overlap = 87.6%; PS = 58.8%); average *d* = 0.20, CI [0.18, 0.23]. These results are depicted in Fig. 1 and described in detail in Supplementary Table 5A.

As in the case of GM_PCP_, sex differences in GM_res_ were devoid of any influence of TIV variation. In this dataset, TIV-VOI linear regression analyses yielded r^2^ values ranging from 6.67^–20^ to 1.98^–28^ (uncorrected *p*-values > 0.99 in all cases; Supplementary Table 5B). All slope values were also virtually 0 (absolute values ranging from 1.47^–9^ to 6.88^–15^), and as Fig. 2 shows, they were uncorrelated with the *p*-values (rho = 0.09, p = 0.308) and the size (unstandardized mean difference, rho = − 0.13, p = 0.152; *d* values, rho = − 0.14, p = 0.148) of the sex differences observed in GM_res._

#### Summary

The results obtained make it possible to draw three main conclusions: First, as previously described^8,10,13,14,18,19^, raw GM_VOL_ conflate sex and TIV variation effects, resulting in large differences that invariably favor the sex with larger TIV (males). Second, as previously shown^8,10,14–16,19,42^, not all the currently used methods are equally effective in statistically removing TIV effects because sex differences calculated using scaling and proportions-adjusted data are still partially due to TIV variation. Accordingly, these two methods are increasingly viewed as suboptimal TIV-adjustment methods^14,16,19,42,43^. Third, when TIV variation is properly controlled, sex differences appear to be bidirectional, and their size is very much reduced, approaching zero in many cases. This last observation also agrees with those of previous studies^10,13,16,18,19,44^.

### Replication of univariate sex differences

Despite being similar to those of other studies, the results described in the previous section do not provide direct information about which sex differences in GM_VOL_ are replicated, or to what extent the replicability of sex differences in GM_VOL_ is affected by different TIV-adjustment methods. In order to answer these two questions, we estimated the 95% prediction intervals for the *d* values of the sex differences in GM_VOL_ previously observed in the UJI sample (Supplementary Table 6A), and we calculated the replication rates observed in each dataset.

As summarized in Table 1, the number of replicated effects greatly differed for raw (52; 44.83%), scaling (72; 62.06%), proportions- (86; 74.14%), PCP- (110; 94.82%) and residuals- (106; 91.38%) adjusted data (χ^2^ = 102.77, df = 4, *p*-value < 2.2^–16^, see pairwise comparisons in Table 1 and Supplementary Table 6B). Based on these results, it is clear that replication rates were higher for those datasets in which TIV variation had been properly controlled than for those in which it had not. Moreover, this effect was observed even though the sex differences in TIV obtained in the HCP sample fell within the prediction interval of the difference observed in the UJI sample (see Supplementary Table 6A). Therefore, it might be tentatively concluded that by controlling the effects of TIV (which can vary in different samples), the PCP and residuals methods provide not only TIV-independent but also more replicable estimates of sex differences in GM_VOL_. However, because this is the first time such an effect has been described, this conclusion requires verification by future independent studies.

**Table 1:** Number and description of replicated effects. The first row of the table provides the number of replicated effects (those Cohen’s *d* values of the HCP sample falling within the prediction interval of their counterparts in the UJI sample). Superscripted letters (A to E) denote a statistically significant different proportion of replicated effects from what was observed in the raw, scaling, proportions, PCP, or residuals datasets, respectively. Effects were designated as “differences” if the 95% CI of their mean difference did not include the zero value and as “no-differences” if they did. Brain areas showing the largest and smallest sex differences are reported along with M > F and F > M averaged Cohen’s d values and their corresponding percent of overlap (o) and superiority (PS). More detailed information about these comparisons and outcomes is provided in Supplementary Table 6.

	Raw (A)	Scaling (B)	Proportions (C)	PCP (D)	Residuals (E)
Number of replicated effects	52 ^B, C, D, E^	72 ^A, D, E^	86 ^A, D, E^	110 ^A, B, C^	106 ^A, B, C^
Differences	52	63	49	47	51
No-differences	0	9	37	63	55
Differences M > F	52	2	6	22	22
Differences F > M	0	56	43	25	29
Differences M > F *d* maximum	1.51 Amygdala R	0.16 Cingulum post_R	0.30 Cingulum post_R	0.31 Pallidum R	0.26 Cerebellum 4_5_R
Differences M > F *d* minimum	0.40 Cerebellum 10_L	0.13 Olfactory R	0.13 Occipital inf_R	0.12 Hippocampus R	0.12 Vermis 9
Differences M > F *d* average	0.96 O: 63.09% PS: 75.16%	0.14 O: 94.42% PS: 53.94%	0.20 O: 92.03% PS: 55.62%	0.18 O: 92.83% PS: 55.06%	0.18 O: 92.83% PS: 55.06%
Differences F > M *d* maximum	–	− 0.80 Hippocampus R	− 0.66 Thalamus L	− 0.30 Frontal_sup Orb_R	− 0.30 Frontal_sup Orb_R
Differences F > M *d* minimum	–	− 0.15 Fusiform R	− 0.13 Rolandic Oper_R	− 0.12 Frontal Sup_R	− 0.11 Precentral R
Differences F > M *d* average	–	− 0.39 O: 84.54% PS: 60.86%	− 0.31 O: 87.64% PS: 58.70%	− 0.18 O: 92.83% PS: 55.06%	− 0.17 O: 93.15% PS: 55.84%

**Figure 3:**
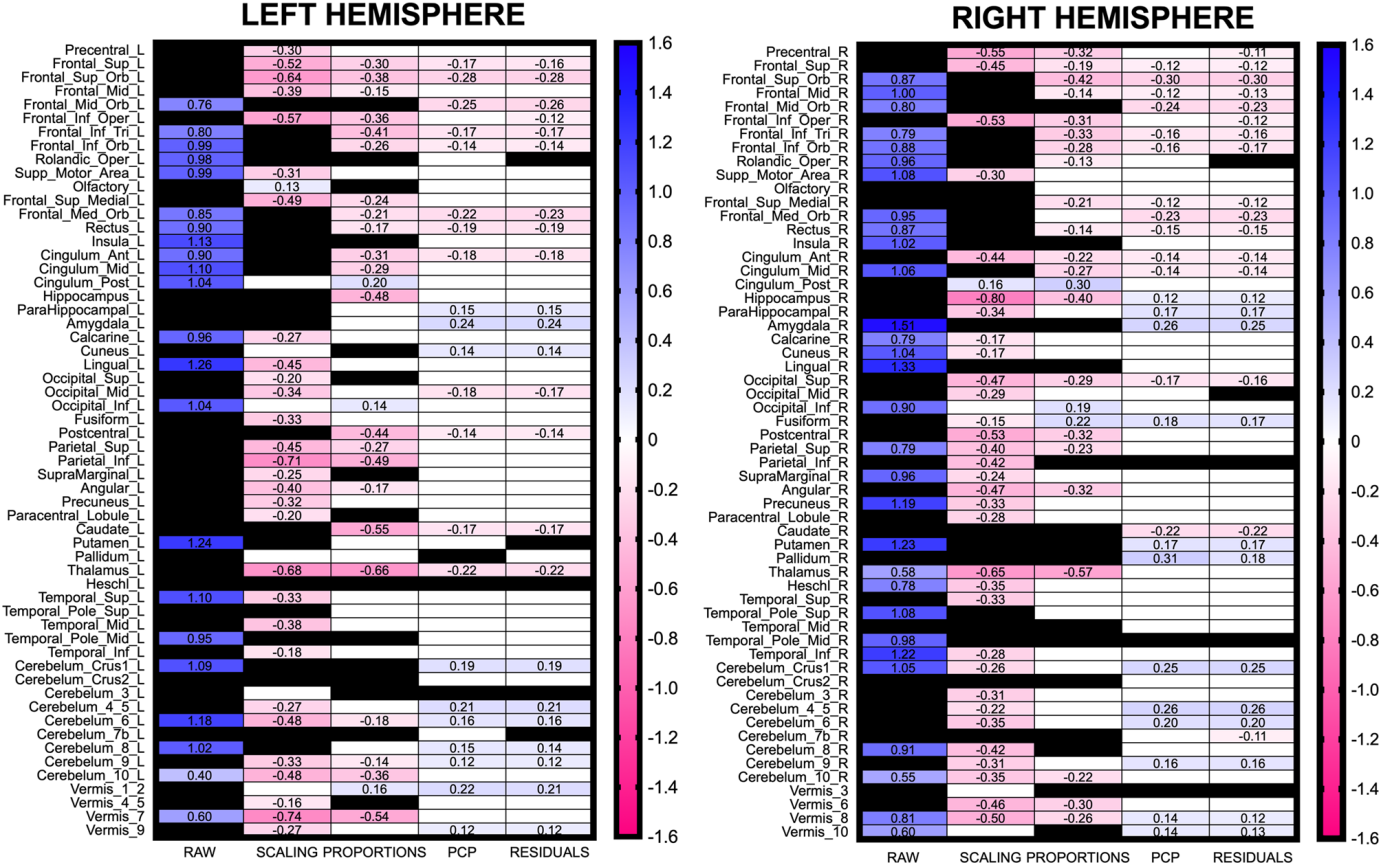
Averaged Cohen’s *d* values for replicated sex differences. As in Fig. 1, panels left and right present odd- and even-numbered brain anatomical regions of the AAL atlas. Heatmaps display the averaged Cohen’s *d* values for replicated sex differences in each dataset, with red colored cells and negative *d* values corresponding to F > M effects, and blue colored cells and positive *d* values corresponding to M > F effects, respectively. Replicated “no-differences” and non-replicated effects are depicted as cells colored in white or black, respectively. An effect was considered to be satisfactorily replicated if its *d* value in the HCP sample fell within the 95% prediction interval of the same effect in the UJI sample (see *Replication of univariate sex differences* in the Materials and Methods section, and Supplementary Table 6 for further details).

The anatomical locations of replicated and non-replicated effects are depicted in Fig. 3. This figure displays the averaged *d* values for each replicated effect whose 95% confidence interval did not include the zero value (referred to as “sex differences” in Table 1). In the same figure, replicated effects whose CIs included the zero value are depicted as white cells (and referred to as “no-differences” in Table 1), but their values and corresponding CIs can be found in Supplementary Table 6C. Non-replicated effects are depicted as black colored cells. For all replicated effects, new prediction intervals estimating the range of *d* values that could be expected in future replication studies assessing sex differences in GM in these anatomical regions were also calculated (Supplementary Table 6D).

From the averaged *d* values calculated for replicated effects, it can again be concluded that in properly TIV-adjusted data, sex effects in local GM_VOL_ are bidirectional and “small” (average |*d*| $\approx$ 0.12; average Overlap $\approx$ 95%; average PS $\approx$ 53.5%). Conversely, when TIV variation is not adequately controlled, sex differences appear much larger in size, and their direction is skewed, either in favor of males (raw data) or females (scaling and proportions datasets).

### Multivariate classification

**Figure 4:**
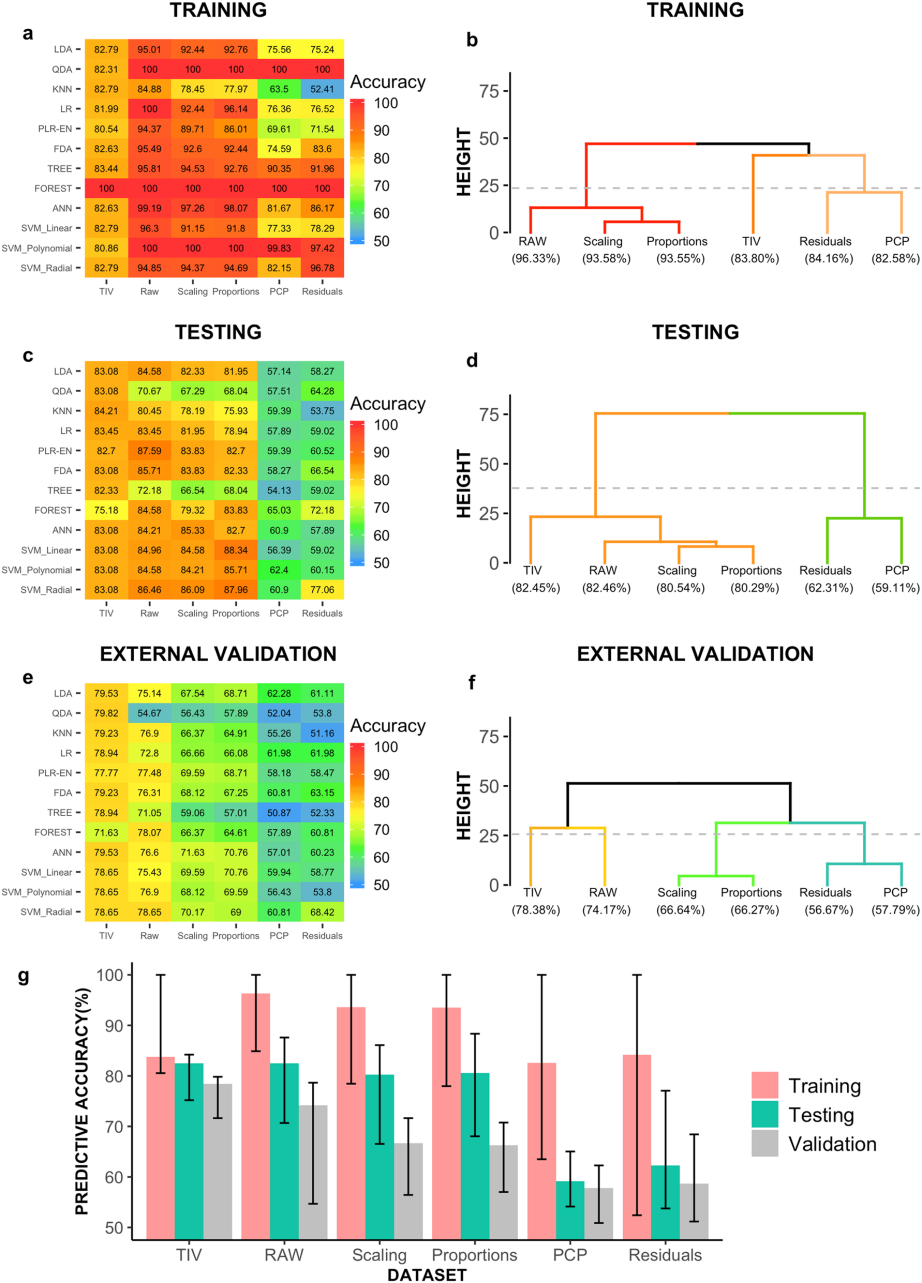
Sex prediction accuracy. The heatmaps depicted in panels a, c, and e show the accuracy rate (percent of correctly predicted cases) for sex prediction obtained by each classifier (rows) in each dataset (columns) in the training, testing, and external validation subsamples, respectively (see *Multivariate classification* in the Materials and Methods section, and Supplementary Table 7 for further details). Note that, in these panels, the order of the rows and columns is constant but arbitrary. The dendrograms depicted in panels b, d, and f display the hierarchical relationships (average linkage based on Euclidean distances) between the prediction results obtained in the TIV, raw, scaling, proportions, PCP, and residuals datasets and their aggregation into performance-based clusters in the training, testing, and external validation subsamples, respectively (see *Multivariate classification* in the Materials and Methods section, and Supplementary Table 8 for further details). In these panels, average performance is reported as bracketed numbers under the dataset labels, and the dashed horizontal line indicates 50% of the maximum height of each dendrogram. Panel g summarizes all the previous results by showing the average (bars) and the maximum and minimum (“whiskers”) of the prediction accuracy observed in each dataset in each subsample.

Figure 4 depicts the classification accuracy achieved by each classifier in the training, testing, and external validation subsamples of the raw, scaling, proportions, PCP, and residuals datasets (for more detailed output, see Supplementary Table 7). As panels A and B of the same figure show, average classification accuracy rates were high (> 80%) in the training subsamples of all the datasets. However, a finer-grained evaluation with the PAM clustering algorithm revealed three distinguishable patterns of results (see Supplementary Table 8). The first cluster was composed of the raw, scaling, and proportions datasets, which exhibited the highest accuracy levels and a high degree of homogeneity, with almost all the classifiers yielding a perfect or nearly perfect (> 90%) classification of females and males. The second cluster consisted solely of the TIV dataset, and it exhibited a large degree of homogeneity, but slightly lower accuracy levels ($\approx$ 84%). Finally, the third cluster was composed of the PCP and the residuals datasets, which exhibited an average accuracy similar to what was observed in the second cluster, but with a much larger variation in the classifiers (range: 52.41–100%).

As could be expected, accuracy rates in the testing subsample were lower than in the training subsample (Fig. 4, panel C), thus revealing that the very high accuracy rates observed in the training subsamples were partly due to overfitting. This decrease was more pronounced when using the local GM_VOL_ of the raw (− 13.87%), scaling (− 13.28%), proportions (− 13.01%), PCP (− 23.46%), and residuals (− 21.85%) datasets as multivariate predictors than when using TIV as a single predictor (− 1.34%). Consequently, the results observed in the testing subsamples were ordered differently from in the training subsamples (entanglement: 25.9%; Supplementary Table 8), now showing only two very clearly separated clusters (see Fig. 4, panel D). The first cluster was composed of all the datasets that incorporated variation due to gross morphology differences between males and females (TIV, raw, scaling, and proportions), and it was characterized by high (> 80%) classification accuracy rates. Conversely, the second cluster, composed of those datasets that were free of any influence of TIV-variation (PCP and residuals), showed much lower ($\approx$ 60%) accuracy rates. It should be noted that accuracy rates showed a wide variation among the classifiers in both clusters. Nevertheless, differences between clusters were evident, even when considering the results obtained with each classifier separately.

As could be expected^34^, accuracy rates were slightly lower in the external validation than in the testing subsample (Fig. 4, panel E). This decrease was larger in the proportions (− 14.02%), scaling (− 13.9%) and raw (− 8.29%) datasets than in the TIV (− 4.07%), residuals (− 3.46%), or PCP (− 1.32%) datasets. This differential reduction in the predictive accuracy did not substantially change the datasets’ ordering (entanglement: 5.75%, see tanglegram in Supplementary Table 8), but it divided the homogeneous cluster 1 from the testing subsample into 3 clusters, while leaving cluster 2 unaffected. Thus, in the external validation subsample, 4 clusters were observed: TIV > Raw > scaling $\approx$ proportions > Residuals $\approx$ PCP (Fig. 4, panel F). In the first cluster (TIV dataset), average accuracy was 78.38%, and all the classifiers yielded very similar prediction accuracy rates (range 71.63–79.82%). In the raw dataset (cluster 2), most of the methods performed slightly worse than in cluster 1, but the poor accuracy exhibited by the QDA classifier (54.67%) was mainly responsible for its slightly lower average accuracy (74.17%). The average accuracy of the third cluster (scaling and proportions datasets) was around 66%, with the QDA and simple classification trees clearly performing below this average. Finally, the fourth cluster was composed of the PCP and residuals datasets, which once again exhibited the lowest average accuracy rates (around 58%), with the QDA, KNN, and simple classification trees showing almost chance-level performance.

These results (summarized in Fig. 4, panel G) confirm and extend those of other previously published studies. In this regard, Chekroud and collaborators ^45^ obtained 92% (CI: 88.9–94.5%) accuracy when predicting sex in a large cohort of young females and males (training subsample, n = 1,156, testing subsample = 400) through an elastic net -penalized logistic regression (P_LR_-EN) that incorporated TIV-uncorrected subcortical GM volumes and cortical thickness measurements as predictors. Similarly, using P_LR_-EN and a support vector machine with a radial kernel (SVM_radial_) as classifiers and a complex array of predictors (raw GM density estimates, scaled GM volumes, age, and intellectual quotient), Anderson and co-workers^37^ found classification accuracy rates above 90% when predicting sex in large cohorts of incarcerated (training subsample, n = 930; testing subsample, n = 370) and non-incarcerated (training subsample, n = 922; testing subsample, n = 526) individuals. These reported accuracy rates are similar but slightly higher than those observed with the same classifiers in the raw dataset of the present study, a fact that is probably related to the use of larger training samples^33,46^ and other procedural discrepancies (e.g. different predictors). However, when considered together, the results of these two preceding studies and our results confirm that sex might be very accurately predicted by TIV-uncorrected GM features.

We also observed that sex prediction accuracy becomes much lower when TIV-variation on local GM_VOL_ is appropriately controlled, but this is not as true when non-appropriate TIV-adjustment methods are employed (Fig. 4). To our knowledge, no previous study had been specifically designed to address this question. However, some reports had provided scattered evidence suggesting that the accuracy of sex prediction is reduced when using appropriate TIV-adjustment methods. Thus, Joel and co-workers reported that an anomaly detector algorithm discriminated between the brains of females and males better when brain features were not corrected for TIV-variation than when the same features were corrected with the PCP method^36^. Similarly, in their previously mentioned study^45^, Chekroud and collaborators observed that the sex prediction accuracy of their P_LR_-EN dropped from 92 to 70% (CI: 65.0–74.2%) when TIV-variation was “regressed out”. On the other hand, two other reports have provided estimates of sex prediction accuracy when using as predictors the same 116 scaled GM_VOL_ that were employed in the present study. Thus, when re-analyzing some previously published data^47^, Rosenblatt found that a SVM_linear_ correctly predicted sex in about 80% of the cases^35^. With the same data, DelGiudice et al. (2015) found that principal component analysis combined with LDA properly predicted the individuals’ sex in about 70% of the cases. All these results are again very similar to those obtained in the present study, and they confirm that the method chosen to control TIV-variation has a major impact on sex predictability.

In summary, based on the results of this and other preceding studies, it can be concluded that sex can be appropriately predicted from raw GM local brain volumes. However, as also occurs when considering univariate sex differences in GM_VOL_ in isolation, the distinctiveness of the brains of females and males at the multivariate level is very much dependent on their gross morphological differences (operationalized here in terms of TIV variation). Indeed, when using TIV as a single predictor, sex might be inferred with approximately the same accuracy and slightly less variance as when 116 raw GM local volumes are used. Conversely, when TIV variation is completely ruled out, the multivariate distinctiveness of the brains of females and males is very much reduced, and high misclassification rates are observed.

## Limitations

The present study has some limitations that should be considered. Some of these limitations are related to the samples used. First, it should be noted that the present study used two convenience samples, rather than random samples obtained through optimized epidemiological procedures. Moreover, the samples used covered a relatively narrow age range. Thus, although these limitations are common in non-clinical brain volumetric studies, the representativeness of conveniences samples is not fully guaranteed, and the results obtained may have limited generalizability, especially for much younger or older populations. Second, although these samples could be considered “large” and ensured the necessary sensitivity to evaluate univariate sex differences, they resulted in case/variable ratios that might be suboptimal for some of the multivariate analyses included in the present study.

In addition, one of the objectives of the present study was to provide a direct replication of the results obtained in a previous study by our team on univariate sex differences in GM_VOL_^19^. This goal constrained some methodological decisions, including the use of a VOI-based approach and, particularly, the AAL atlas. The use of predefined VOIs has several advantages (e.g. avoids circularity, reduces the number of between-group comparisons,…), and it contributes to more accurate estimation of effect sizes^48,49^. However, the use of any predefined template (and that of the AAL atlas in particular) reduces anatomical precision and introduces other limitations and challenges^50,51^ that without compromising the validity of the present results, impede a direct comparison with estimates of GM_VOL_ sex differences obtained with voxel-wise approaches.

Finally, the present study explored the use of the local GM_VOL_ in predicting sex as a feasible approach to assess the degree of multivariate distinctiveness of male and female brains. In this attempt, the performance of 12 classification algorithms with distinct statistical assumptions and intrinsic operations was evaluated. However, even this ample exploration does not exhaust all the possible methods, and different results could be obtained with other classification algorithms, other predictors, or different parameters of the classifiers tested in the present study. This limitation does not reduce the validity of our results and conclusions about the effects of different TIV-adjustment methods on sex prediction. However, this limitation suggests that additional caution is needed when using the prediction accuracy rates obtained in this study as estimates of the multivariate morphological distinctiveness of the brains of females and males.

## Conclusion

Our results show that univariate and multivariate sex differences in GM_VOL_ are largely dependent on male–female differences in TIV, and that when this source of variation is parsed out univariate and multivariate sex differences are very much reduced. Our results also show that not all currently used TIV-adjustment methods are equally effective to remove TIV variation, and that which method is finally used has a major impact on the size (and, in the case of univariate differences, also the direction and, probably, the replicability) of the estimated sex differences. Consequently, choosing an appropriate TIV-adjustment method becomes a critical methodological decision that should be carefully considered and explicitly reported when designing new studies or when summarizing/ meta-analyzing preceding results.

## Materials and methods

### Participants

This study was conducted using data from two samples. The “HCP-sample” was composed of 444 females an 444 males included in the 1,200 Subject Release of the Human Connectome Project (HCP) ^52^, who did not differ in age (Mean_females_ = 28.76, SD = 3.59; Mean_males_ = 28.52, SD = 3.40). On the other hand, the “UJI-sample” ^19^ was composed of 171 females and 185 males with similar ages (Mean_females_ = 22.39, SD: 3.04; Mean_males_ = 21.64, SD: 4.90) See Supplementary Table 9 for further details.

### Imaging data and TIV-adjustment

#### MRI acquisition

The MRI acquisition details of the HCP-sample might be found at the reference manual of the S1200 release of the HCP (https://www.humanconnectome.org/storage/app/media/documentation/s1200/HCP_S1200_Release_Reference_Manual.pdf). The details of the MRI data for the UJI subsample can be found in^19^.

#### Image pre-processing

All images were preprocessed with the VBM8 toolbox (version r445) implemented in the “New Segment” toolbox of the SPM8 (https://www.fil.ion.ucl.ac.uk/spm/software/spm8/) software (version 6316). This protocol includes four main steps: (1) segmentation of the images into gray matter, white matter, and cerebrospinal fluid; (2) registration to a standard template provided by the International Consortium of Brain Mapping (ICBM); (3) a high-dimensional DARTEL normalization of the gray matter segments to the MNI template; and (4) a data quality check (in which no outliers or incorrectly aligned cases were detected). After applying this procedure, which does not include any correction for overall head size, voxels were mapped into 116 regions according to the Automated Anatomical Labeling atlas (AAL, ^50^) by calculating the total gray matter volume for each region of interest (VOI) and participant via a MATLAB script (https://www0.cs.ucl.ac.uk/staff/g.ridgway/vbm/get_totals.m).

On this initial dataset (referred to as “raw”) sex differences unadjusted for TIV-variation were evaluated. Moreover, all the TIV adjustment methods (except the “scaling” method) were applied a posteriori to this initial output to generate TIV-adjusted datasets. On the other hand, TIV was estimated using native-space tissue maps obtained in the VBM8 segmentation step. Briefly, TIV was calculated as the sum of GM, WM and CSF total values multiplied by voxel size and divided by 1,000 to obtain a milliliter (ml) measurement. Although automated TIV estimation is less precise than that obtainable by manual segmentation^43^, this possible bias is not a major concern in the present study that used the same TIV estimation procedure when comparing different TIV-adjustment methods in a large sample of participants.

#### TIV-adjustment methods

Briefly, the four TIV-adjustment methods compared in the present study were:

*–Scaling*: Scaling is a normalization-related option provided in several image processing software packages that intends to remove the effects of head size (TIV) variation in local volumes using a two-step procedure. First, all brains are deformed as to make them to have exactly the same size. Second, the obtained normalized GM segments are multiplied by the non-linear determinants of the normalization deformation matrix. In this way, all GM segments are scaled to have the same size while local differences in volume are preserved. In the present study, the scaling method was implemented by using the non-linear modulation option included in the VBM8 toolbox^53^.

*–Proportions adjustment method* (proportions): This method attempts to provide adjusted VOIs by simply dividing each individual’s unadjusted VOI value by the value of its TIV^8^.

–*The power-corrected proportions method* (PCP): This method was recently proposed^14^ as a way to improve the proportions approach by introducing an exponential correcting parameter (VOI_adj_ = VOI/TIV^b^) in the denominator. This parameter (b) corresponds to the slope value of the LOG(VOI) ~ LOG(TIV) regression line.

–*The residuals method* (residuals): This method was originally described by^27^ and it aims to remove TIV-VOI relationships through the formula VOI_adj_ = VOI − b(TIV − $\stackrel{-}{TIV}$), where b is the slope value of the TIV-VOI regression line, and $\stackrel{-}{TIV}$ denotes the mean of the TIV values for all the participants.

### Statistical analyses

#### Univariate sex differences in the HCP sample

Following current recommendations^54,55^, the statistical analyses focused on estimating effect sizes and 95% confidence intervals (CI) rather than on testing statistical significance.

Standardized effect sizes for between-mean differences (Cohen’s *d*) and their 95% CIs were calculated for each VOI in the raw, scaling, proportions, PCP, and residuals datasets of the HCP-sample. In the present study, positive Cohen *d* values indicate larger GM_VOL_ in males than in females (M > F), whereas negative Cohen *d* values denote larger GM_VOL_ in females than in males (F > M). To facilitate interpretation^29^, *d* values were transformed into the Weitzman’s $\Delta$ (also known as percent of overlap and *ORL-1*) and the percent of superiority (PS). The percent of overlap denotes the proportion of scores that overlap in two normal distributions whose means differ in some magnitude. PS denotes the probability that a randomly sampled member of population *a* will have a score that is higher than the score attained by a randomly sampled member of population *b*^29^.

Following current recommendations^56^, unstandardized effect sizes for sex differences in GM_VOL_ were also calculated. The 95% CIs of these differences were used to identify statistically significant sex differences (e.g. a 95% CI for the difference between two means that includes the zero value makes it possible to reject a nil null hypothesis at p < 0.05^57^). Exact *p*-values were obtained through separate Student’s *t* tests for independent groups. No corrections for multiple comparisons were introduced initially, but FWER and FDR adjusted *p*-values using the Benjamin-Hochberg ^58^ and Bonferroni-Dunn ^59^ methods, respectively, were also calculated (see Supplementary Tables 1A–5A).

Previous studies have shown that raw GM_VOL_ are directly related to TIV^12,14,16,42^, and that the strength of these relationships (slope values of linear TIV-VOI_raw_ regressions) is ordinally correlated (Spearman’s rho) with the size and *p*-values of the sex differences found in these VOI_raw_^19^. Conversely, VOIs adjusted (VOI_adj_) with appropriate methods no longer show a linear relationship with TIV, and the size and *p*-values of the sex differences in GM_VOL_ are uncorrelated with the TIV-VOI_adj_ slope values^19^. Therefore, in the present study, we employed the same regression-based approach to assess the efficacy of each TIV-adjustment method in eliminating the effects of TIV variation.

#### Replication of univariate sex differences

Following current recommendations^23,25^, effects’ replication was assessed by calculating Prediction Intervals (PIs). More specifically, appropriate PIs were calculated to assess to what extent the *d* values obtained in each dataset of the HCP sample replicated those previously observed in the same datasets of the UJI sample^19^. PIs estimate the range of values within which a parameter (e.g., Cohen’s *d* value) would fall in future replication studies if differences among studies were solely due to sampling error^23,25^. Thus, when a replication result falls outside the prediction interval, the results of the original study are not properly replicated, and it can be concluded that factors other than sampling error were operating to produce distinct results in each study.

PIs for the sex differences in GM_VOL_ observed in the UJI sample were calculated with the *predictionInterval* package for R^60^. A second step was to identify whether each of these PIs captured the corresponding *d* value in the HCP sample (see^25^ for details). From these data, the percent of successfully replicated effects (replication rates) in each dataset was estimated and compared to the others. These comparisons were conducted by means of the χ^2^ test for independence, followed by appropriate dyadic comparisons using the pairwise tests of independence for nominal data from the *rcompanion* package for R^61^. All replicated effects were meta-analyzed with the *metafor* package for R^62^, hence obtaining weighted average *d* values and their corresponding CIs. From these new estimates, 95% PIs estimating the range of expected values of *d* at each VOI in possible future replication studies were also calculated (Supplementary Table 6).

#### Multivariate classification

To assess the effects of TIV-adjustment on the utility of the 116 VOIs defined by the AAL atlas in predicting sex categorically defined as male or female, we tested 12 supervised classification algorithms (see below) in the raw, scaling, proportions, PCP- and residuals-adjusted datasets. Moreover, to provide a reference point for judging the results obtained, the same analyses were repeated using TIV as a single predictor of sex. Before being used as predictors, all these variables were transformed into *z*-scores to avoid distortions due to their different ranges^33,63^.

Following current recommendations^33,34^, classification algorithms were fitted, tested, and validated in separate groups of participants with the same number of females and males (hence avoiding classification distortions due to between-class imbalance^64,65^). Thus, each algorithm was initially fitted in a randomly selected *training subsample* (311 females and 311 males) from the HCP-sample, internally validated^34^ in the *testing subsample* (the 133 females and 133 males hold-out participants from the HCP sample) and externally validated^34^ in the so-called *external validation subsample* (171 males and 171 females randomly extracted from the UJI-sample). The classifiers’ performance was primarily evaluated in terms of overall accuracy (percent of correctly classified cases and its 95% CI), although a standardized measure of the concordance between the predicted and actual sex of the participants in each sample (Cohen’s Kappa and its 95% CI) is also provided in Supplementary Table 7.

Instead of relying on the estimates provided by a single classifier, we opted to calculate, report, and compare the prediction accuracy rates obtained with 12 classification methods. It was important to test several methods because the predictive accuracy achieved by a particular classifier is very much dependent on whether or not the data characteristics satisfy the assumptions (e.g. normality, linearity…) under which the classifier operates^33,66^, and these data characteristics are likely to differ across the datasets compared in the present study or across samples from different studies. Described briefly, the classifiers tested were:

##### Linear discriminant analysis (LDA)

LDA has traditionally been the parametric method of reference for classification studies. LDA assumes normality and equality of variances/covariances^33,67^. In the present study, LDA was implemented using the default options of the *rda* function of the *MASS* package for R^68^.

##### Quadratic discriminant analysis (QDA)

QDA is a similar classification method to LDA, but (1) QDA does not assume a common covariance matrix; (2) QDA classification is based on quadratic decision boundaries; (3) QDA is more sensitive to small sample size (or n/ predictor ratios), and it presents greater variance but less bias than LDA^33,69^. In the present study, QDA was implemented using the default options of the *qda* function of the *MASS* package for R^68^.

##### K-nearest neighbors (KNN)

KNN is a simple but often powerful classifier that does not make any assumptions about the data distribution^70^. When K must be kept constant in order to compare several sets of predictors, it is customary to fix K as the square root of the number of subjects included in the training sample^71^. Therefore, in the present study, the K value was pre-established as K = 25 ($\sqrt{622}$ = 24.93), and the KNN classifier was implemented through the *knn* function of the *class* package for R^68^.

##### Logistic regression (LR)

LR was implemented using the *glm* function of the *stats* package for R. LR is a linear classification method similar to LDA, but it does not assume normality, and it is less sensitive to outlier effects, hence outperforming LDA when the normality assumption is severely violated^72^..

##### Penalized logistic regression with an elastic net (P_LR_-EN)

P_LR_-EN was implemented using the *glmnet* function of the glmnet package for R^73^. P_LR_-EN is a form of logistic regression that reduces the number of variables in the regression model by penalizing the coefficients of the variables that contribute less to the prediction, using an “elastic” criterion that sets some of these coefficients to exactly zero while merely shrinking other coefficients toward zero^74^. Compared to traditional LR procedures, P_LR_-EN often (but not always) exhibits reduced bias and increased predictive performance^75^.

##### Flexible discriminant analysis (FDA)

FDA can briefly be described as performing LDA in an enlarged feature space, usually showing much higher predictive accuracy than LDA^33,76^. In the present study, non-penalized FDA was implemented using the *fda* function of the *mda* package^77^, employing the adaptive additive-spline regression function of the *BRUTO* subroutine of this R package.

##### Tree-based classifiers

Classification trees do not make any strong assumptions about the data and they operate by segmenting the feature space into a number of non-overlapping regions through a recursive binary splitting process^33,78^. At the risk of overfitting, accuracy might be enhanced by aggregating a large number of decision trees into a single random forest, each of them using a limited subset of predictors (ordinarily, $\sqrt{p}$). In the present study. a simple classification tree and a complex random forest (500 trees with 10 randomly selected predictors each) were implemented using the *tree* package for R^79^.

##### Artificial neuronal networks (ANN)

ANNs are very powerful but opaque learning algorithms that extract linear combinations of inputs as derived features, which in turn are used to non-linearly model the classification problem^33,80^. In the present study, a simple ANN was constructed by using the default specifications of the *neuralnet* package for R^81^.

##### Support-vector machines (SVMs)

SVMs is a generic name for a series of very flexible procedures that produce nonlinear classification boundaries by constructing linear boundaries into an enlarged feature space using all or just a fraction of the cases^33,82^. In the present study, the *tune* function (tenfold cross-validation) was used to automatically select the optimal values for the regularization (*C*; tested range: from 1^–3^ to 1^3^) and kernel-width (γ; tested range: 0.0001, 0.001, 0.01, 0.1, 0,5, 1, 2, 3, 4, 5) parameters when building the SVMs with linear, radial, and polynomial (degree = 3) kernels, using the *svm* function of the *e1071* package for R^83^.

To identify which datasets exhibited similar predictive performance across methods in the training, testing, and external validation subsamples, a robust outlier clustering method (the partitioning around medoids algorithm; PAM) was applied^84^. Thus, for each subsample, the PAM algorithm of the *cluster* package for R ^85^ was run four times, each time setting the number of clusters (K) to 2, 3, 4, or 5, respectively. The K value that maximized the average silhouette was considered the optimal number of clusters in each subsample (see ^84^ for further details). To provide a graphical representation of the clusters’ composition and the between-cluster dissimilarities in each subsample, three separate dendrograms were constructed with the *dendextend* package for R^86^ by subjecting the accuracy rates obtained in each dataset to a hierarchical cluster analysis (average linkage based on Euclidean distances) and then cutting them at appropriate heights to illustrate the clusters previously identified by the PAM algorithm. Of note, in all cases, between-cluster separation was at least fivefold larger than the average within-cluster dissimilarity, and all the obtained clusters only merged at above 50% of the maximum height of their dendrograms (see Supplementary Table 8). These observations indicate that the identified clusters are not a product of random variation, but rather they correspond to specific/ meaningful predictive performance profiles.

### Ethics approval and consent to participate

This study was carried out in accordance with the recommendations of the ethical standards of the American Psychological Association. The protocol was approved by the Ethics Standards Committees of the Universitat Jaume I. In accordance with the Declaration of Helsinki, all subjects of the HCP and UJI samples gave written informed consent prior to participating.

## Supplementary information

Supplementary Tables

## Data Availability

This study was primarily conducted using data from the open source 1,200 Subject Release (S1200) of the Human Connectome Project (HCP). The access to this sample should be directly requested to the Washington University—University of Minnesota Consortium of the Human Connectome Project (WU-Minn HCP). The second sample used in this study (UJI sample) was kindly provided by Dr. César Ávila of Universitat Jaume I. Requests for accessing this second sample should be directly addressed to, and authorized by, Dr. César Ávila.

## Abbreviations

AAL: Automated anatomical labeling atlas
ANN: Artificial neuronal net
CI: Confidence interval
F: Females
FDA: Flexible discriminant analysis
FDR: False discovery rate
FWER: Family wise error rate
GM: Gray matter
GM_PCP_: Gray matter volumes adjusted with the power-corrected-proportions’ method
GMprop: Gray matter volumes adjusted with the proportions’ method
GM_raw_: Gray matter volumes of the raw dataset
GM_res_: Gray matter volumes adjusted with the residuals’ method
GM_VOL_: Gray matter volume
HCP: Human connectome project
KNN: K-nearest neighbors
LDA: Linear discriminant analysis
LR: Logistic regression
M: Males
MRI: Magnetic resonance imaging
PAM: Partitioning around medoids algorithm.
PCP: Power-corrected proportions
PI: Prediction interval
PLR-EN: Penalized logistic regression (elastic net)
PS: Percent of superiority
QDA: Quadratic discriminant analysis
SVM: Support vector machine
TIV: Total intracranial volume
VBM: Voxel based morphometry
VOI: Volume of interest
VOI_adj_: Adjusted VOI
