# Author Correction: Effects of different intracranial volume correction methods on univariate sex differences in grey matter volume and multivariate sex prediction

**DOI:** 10.1038/s41598-020-75522-7

**Published:** 2020-10-29

**Authors:** Carla Sanchis-Segura, Maria Victoria Ibañez-Gual, Naiara Aguirre, Álvaro Javier Cruz-Gómez, Cristina Forn

**Affiliations:** 1 Departament de Psicologia Bàsica, Clínica i Psicobiologia, Universitat Jaume I, Avda. Sos Baynat, SN, 12071 Castelló, Spain; 2 Department of Mathematics, IMAC, Universitat Jaume I, Castelló, Spain

Correction to: *Scientific Reports* https://doi.org/10.1038/s41598-020–69361-9, published online 31 July 2020.

The original version of this Article contained a typographical error in the spelling of the author Álvaro Javier Cruz-Gómez.

Consequently, this Article also contained an error in the Contributions section.

“A.J.G.-C. processed the scan images”.

now reads:

“A.J.C.-G. processed the scan images”.

These errors have now been corrected in the HTML and PDF versions of this Article.

